# International Clinic

**Published:** 1892-08

**Authors:** 


### Book Reviews.

INTERNATIONAL CLINICS.—Edited by John M. Keating, M. D., Colorado Springs, Col.; Judson Daland, M. D., Philadelphia; J. Mitchell Bruce, M. D., F. R. C. P., London, Eng.; and David W. Finlay, M. D., F. R. C. P., Aberdeen, Scotland. Vol. I, Second series, 1892. J. B. Lippincott Co., Philadelphia.

THIS publication, just entering its second year, presents its usual neat appearance and an array of contributors that bespeaks for it the high character of its contents. The articles, as heretofore, are divided into the general headings of Medicine, Neurology, Pediatrics, Surgery, Genito-Urinary and Venereal Diseases; Gynecology and Obstetrics, Ophthalmology, Laryngology and Dermatology, and is altogether very conveniently arranged. We bespeak for the second series the popularity and ready sale which so distinguished the first. C. E. J.

